# Interfamily Transfer of Dual NB-LRR Genes Confers Resistance to Multiple Pathogens

**DOI:** 10.1371/journal.pone.0055954

**Published:** 2013-02-20

**Authors:** Mari Narusaka, Yasuyuki Kubo, Katsunori Hatakeyama, Jun Imamura, Hiroshi Ezura, Yoshihiko Nanasato, Yutaka Tabei, Yoshitaka Takano, Ken Shirasu, Yoshihiro Narusaka

**Affiliations:** 1 Research Institute for Biological Sciences Okayama, Okayama, Japan; 2 Graduate School of Life and Environmental Sciences, Kyoto Prefectural University, Kyoto, Japan; 3 Vegetable Breeding and Genome Research Division, NARO Institute of Vegetable and Tea Science, Mie, Japan; 4 Graduate School of Agriculture, Tamagawa University, Tokyo, Japan; 5 Faculty of Life and Environmental Sciences, University of Tsukuba, Ibaraki, Japan; 6 Genetically Modified Organism Research Center, National Institute of Agrobiological Sciences, Ibaraki, Japan; 7 Graduate School of Agriculture, Kyoto University, Kyoto, Japan; 8 RIKEN Plant Science Center, Yokohama, Japan; Virginia Tech, United States of America

## Abstract

A major class of disease resistance (*R*) genes which encode nucleotide binding and leucine rich repeat (NB-LRR) proteins have been used in traditional breeding programs for crop protection. However, it has been difficult to functionally transfer NB-LRR-type *R* genes in taxonomically distinct families. Here we demonstrate that a pair of *Arabidopsis* (Brassicaceae) NB-LRR-type *R* genes, *RPS4* and *RRS1*, properly function in two other Brassicaceae, *Brassica rapa* and *Brassica napus,* but also in two Solanaceae, *Nicotiana benthamiana* and tomato (*Solanum lycopersicum*). The solanaceous plants transformed with *RPS4*/*RRS1* confer bacterial effector-specific immunity responses. Furthermore, *RPS4* and *RRS1,* which confer resistance to a fungal pathogen *Colletotrichum higginsianum* in Brassicaceae, also protect against *Colletotrichum orbiculare* in cucumber (Cucurbitaceae). Importantly, *RPS4*/*RRS1* transgenic plants show no autoimmune phenotypes, indicating that the NB-LRR proteins are tightly regulated. The successful transfer of two *R* genes at the family level implies that the downstream components of *R* genes are highly conserved. The functional interfamily transfer of *R* genes can be a powerful strategy for providing resistance to a broad range of pathogens.

## Introduction

Plants trigger innate immune responses to pathogens via a two-layer surveillance system composed of pattern recognition receptors (PRRs) and nucleotide binding-leucine rich repeat (NB-LRR) proteins that are encoded by resistance (*R*) genes [1]. PRRs recognize microbe-associated molecular patterns (MAMPs) at an plasma membrane, and NB-LRR proteins subsequently detect pathogen-derived effectors inside the cell. The two layers are often called MAMPs-triggered Immunity (MTI) and effector-triggered immunity (ETI) [2]. Because MAMPs are conserved across a wide range of microbes, transfer of PRRs could confer resistance to a broad range of plant pathogens [3]. Likewise, some effectors are conserved within groups, so the transfer of NB-LRR proteins could confer resistance to phytopathogens carrying the common effectors. Furthermore, NB-LRR-based ETI is generally much stronger than PRR-mediated MTI, and thus NB-LRR transfer could be a powerful tool for disease control. *R* genes that confer NB-LRR-based ETI have been introgressed from wild relatives into susceptible varieties of the same species in traditional breeding programs. However, it has been difficult to transfer NB-LRR-type *R* genes to confer disease resistance in a different family [4]. Heterologous expression of NB-LRR-type *R* genes in a taxonomically distinct family often triggers either no response or inappropriate autoimmunity responses, suggesting that the regulatory or signaling components associated with NB-LRR protein-based resistance are potentially family specific [5]. There are, however, examples in which transient expression of particular *R* genes triggers correspoonding effetor dependent responses [6]. To date, stable transformaion with NB-LRR-type *R* genes in different families to confer actual resistance to pathogens has not been reported.

**Figure 1:**
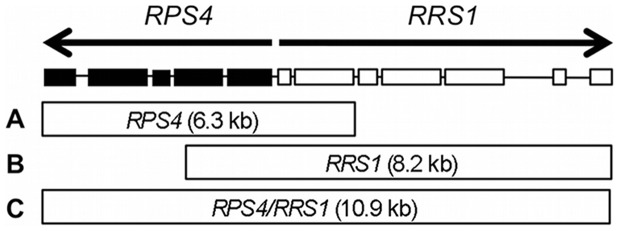
Schematic diagram of *RPS4* and *RRS1*. *RPS4* and *RRS1* genes are arranged in a head-to-head configuration in *A. thaliana* ecotype Wassilewskija chromosome V. Because the two open reading frames are only 264 bp apart, the promoter regions are likely to be overlapping, leading to co-regulation of the genes. Exons of *RPS4* and *RRS1* are indicated by black and white boxes, respectively. Intron positions are indicated as lines. (A) The 6.3 kbp genomic *RPS4* fragment, including approximately 2.1 kbp upstream and 109 bp downstream regions, (B) The 8.2 kbp genomic *RRS1* fragment, including approximately 1.8 kbp upstream and 176 bp downstream regions, (C) The 10.9 kbp genomic fragment containing both *RPS4* and *RRS1.*

In a previous study we demonstrated that *Arabidopsis thaliana* (*A. thaliana*) (Brassicaceae) NB-LRR-type *R* genes *RPS4* and *RRS1* function together to confer resistance against multiple pathogen isolates [7], *i.e*., the fungal pathogen *Colletotrichum higginsianum* (*C. higginsianum*), and two taxonomically distinct bacteria, *Pseudomonas syringae* pv. *tomato* DC3000 carrying the effector AvrRps4 (*Pst-avrRps4*) [8] and *Ralstonia solanacearum* (*R. solanacearum*) strains, which express the PopP2 effector [9]. *RPS4* and *RRS1* are encoded in a head-to-head configuration within the *Arabidopsis* genome (Figure 1). As the two open reading frames are only 264 bp apart, the promoter regions of the gene pairs likely overlap, leading to the co-regulation [7], [10]. *RPS4* encodes an NB-LRR with a TIR domain at the N terminus (TIR-NB-LRR), whereas *RRS1* encodes a TIR-NB-LRR with a WRKY domain at the C-terminus. The precise mechanism of how RPS4 functions with RRS1 is not clear.

The goal of this study is to determine whether the dual *R* genes, *RPS4* and *RRS1*, function as a cassette and therefore could confer disease resistance to different plant species. We show that a genomic fragment containing *RPS4* and *RRS1* under the control of their native promoters confers resistance to fungal and bacterial pathogens in the Brassicaceae, Solanaceae and Cucurbitaceae plants. We also demonstrate that the dual *R* gene transgenic plants do not trigger inappropriate auto-immunity responses. These results indicate that the dual *R* gene pair can confer multiple pathogen resistance in taxonomically distinct families.

## Results

### RPS4 and RRS1 Confer Resistance to C. higginsianum in Transgenic Brassica Rapa and Brassica Napus

**Figure 2:**
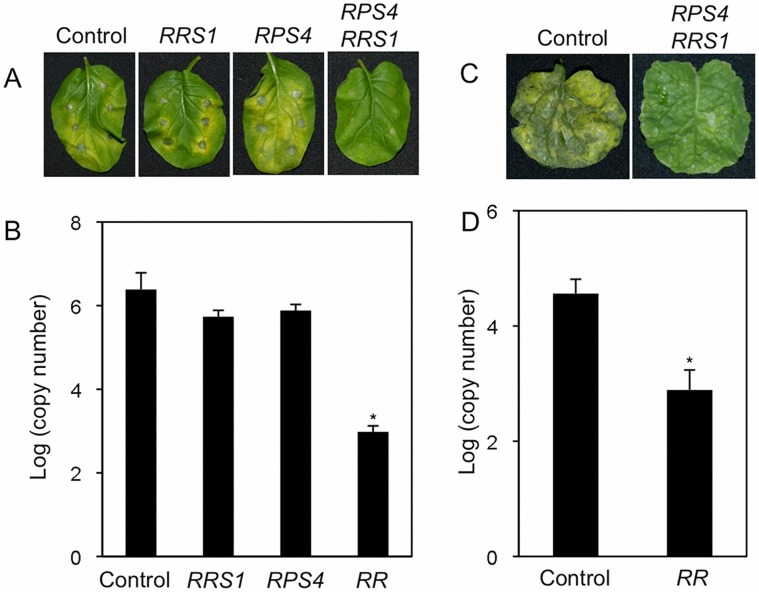
*Colletotrichum higginsianum* resistance analysis in transgenic *Brassica* plants expressing *RPS4* and *RRS1*. (A) Infection phenotypes of *B. rapa* plants leaves inoculated with *C. higginsianum*. Four, three, and one independent T2 transgenic *B. rapa* lines carrying both *RPS4* and *RRS1* (*RR*), either *RRS1* or *RPS4* alone, respectively, were tested. Mature leaves of 2.5 true leaf stage seedlings were inoculated with spotting 5 µl of a conidial suspension of *C. higginsianum* (5×10^5^ spores ml^−1^) on the leaf. Photographs were taken at 6 dpi. Each picture shows a representative of three independent experiments. (B) Quantification of *C. higginsianum in planta* by qRT-PCR. Mature leaves of 2.5 true leaf stage seedlings were spray-inoculated with a conidial suspension of *C. higginsianum* (5×10^5^ spores ml^−1^). Inoculated *B. rapa* leaves were harvested at 4 dpi, and total RNA was isolated. QRT-PCR was performed with *C. higginsianum actin* (*Chin-ACT*) primers for each sample. (C) Infection phenotypes of *B. napus* plants leaves inoculated with *C. higginsianum*. Four independent T2 transgenic lines carrying both *RPS4* and *RRS1* (*RR*) were tested. Mature leaves of 2.5 true leaf stage seedlings were spray-inoculated with a conidial suspension of *C. higginsianum* (5×10^5^ spores ml^−1^). Photographs were taken at 6 dpi. Each picture shows a representative of three independent experiments. (D) Quantification of *C. higginsianum in planta* by qRT-PCR. Inoculated *B. napus* leaves were harvested at 4 dpi, and total RNA was isolated. QRT-PCR was performed with *Chin-ACT* primers for each sample. Bars indicate SE. The asterisks indicate statistical significance from the WT controls (Dunnett’s method, *P*<0.05). This experiment was repeated three times with similar results.

*Colletotrichum* sp. are ascomycete fungi causing anthracnose diseases on a large number of agronomically important crops and vegetables. *C. higginsianum* causes anthracnose disease in Brassicaceae plants. To investigate whether the *RPS4* and *RRS1* pair functions in other Brassicaceae plants, we generated transgenic *Brassica rapa* (*B. rapa*) L. Perviridis Group (Japanese Mustard Spinach, Komatsuna) and *Brassica napus* (*B. napus*) L. plants expressing either *RPS4* or *RRS1*, and *RPS4* and *RRS1* together under the control of the native promoter (Figure 1). *Agrobacterium*-mediated transformation produced four *B. rapa* primary transformants (T1) carrying both *RPS4* (6.3 kbp) and *RRS1* (8.2 kbp) genomic fragments under control of the native *Arabidopsis* promoters. Seventeen *B. rapa* T1 plants carrying only *RRS1* genomic fragment, but only one for *RPS4* were obtained. We also obtained four *B. napus* T1 plants carrying a 10.9 kbp genomic fragment containing *RPS4* and *RRS1*. Secondary transformants (T2) derived from bud pollination of *B. rapa* and selfing *B. napus* T1 plants were assessed for the presence of the transgenes by PCR (Figure S1) (data not shown). Expression of the transgenes in T2 plants was confirmed by qRT-PCR (Figure S2). When inoculated with *C. higginsianum*, wild-type (WT) *B. rapa* and *B. napus* plants developed the brown necrotic lesions typical of anthracnose. *B. rapa* and *B. napus* plants transformed with the *RPS4* and *RRS1* pair were highly resistant to the pathogen, developing only small necrotic flecks at the inoculated sites (Figure 2A–D). However, transgenic *B. rapa* plants expressing either *RPS4* or *RRS1* alone were as susceptible to *C. higginsianum* as WT (Figure 2A, B). These results indicate that *RPS4* and *RRS1* are both needed to confer resistance to the pathogen in Brassicaceae. Transgenic plants expressing *RPS4* and *RRS1* grew normally and did not constitutively express inducible defense gene *PR1*, suggesting that no autoimmunity response was induced by introducing the *R* genes (Figure S3A, B & S4).

### *Nicotiana benthamiana* Plants Expressing *RPS4* and *RRS1* Recognize Bacterial Effectors AvrRps4 and PopP2

**Figure 3:**
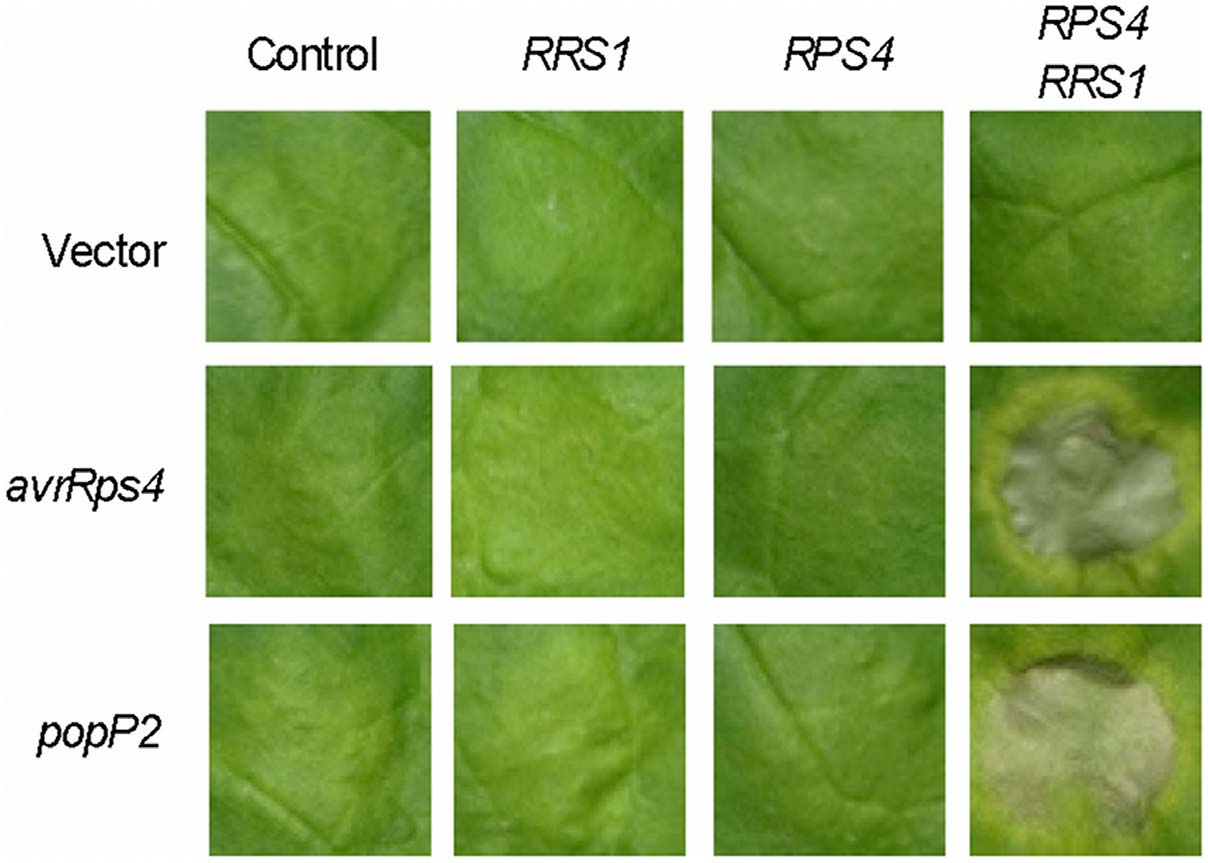
Transient expression assay in *Nicotiana benthamiana* transformed with *RPS4* and/or *RRS1*. Transient expression assay of *avrRps4* or *popP2* was performed by *Agrobacterium* infiltration in four-week-old T3 homozygous transgenic *N. benthamiana* leaves expressing *RPS4* and/or *RRS1*. Two, one, and two independent T2 transgenic lines carrying both *RPS4* and *RRS1*, either *RRS1* or *RPS4* alone, respectively, were tested. Photographs were taken at 10 dpi.

Because *RPS4* and *RRS1* function properly together in three different Brassicaceae plant species, we thought it is possible that this *R* gene pair would be able to break the restricted taxonomic functionality boundary when expressed in non-Brassicaceae plants. We generated transgenic *Nicotiana benthamiana* (*N. benthamiana*) (Solanaceae) plants expressing *RPS4* and *RRS1* under the control of their cognate promoters. We obtained thirteen *N. benthamiana* T1 plants carrying a 10.9 kbp genomic fragment containing *RPS4* and *RRS1* and two with only *RPS4* and one with only *RRS1.* T2 and T3 progenies derived from selfing T1 and T2 plants, respectively, were assessed for the presence of the transgenes by PCR (Figure S1) and T3 homozygous lines were used in all assays described here. Expression of the transgenes in T3 plants was confirmed by qRT-PCR (Figure S2). To test functionality of the *R* genes, we used AvrRps4 or PopP2 effectors which are specifically recognized by the *RPS4* and *RRS1* pair in *Arabidopsis*. We found that AvrRps4 or PopP2 produced by *Agrobacterium*-mediated transient expression under control of a cauliflower mosaic virus (CaMV) 35S constitutive promoter induced cell death in *N. benthamiana* transformed with *RPS4* and *RRS1*, but not in plants expressing only *RPS4* or *RRS1,* indicating that the *R* gene pair is able to recognizes the AvrRps4 or PopP2 effectors in a non-Brassicaceae plant (Figure 3). We verified by qRT-PCR quantification that mRNAs for *avrRps4* and *popP2* accumulated to similar levels in transgenic plants. (Figure S5). No inappropriate autoimmune responses were induced by the introduced *R* gene pair in transgenic *N. benthamiana* plants (Figure S3C & S4).

### Transgenic Tomato Plants Expressing *RPS4* and *RRS1* Confer Resistance to Two Taxonomically Distinct Bacteria

**Figure 4:**
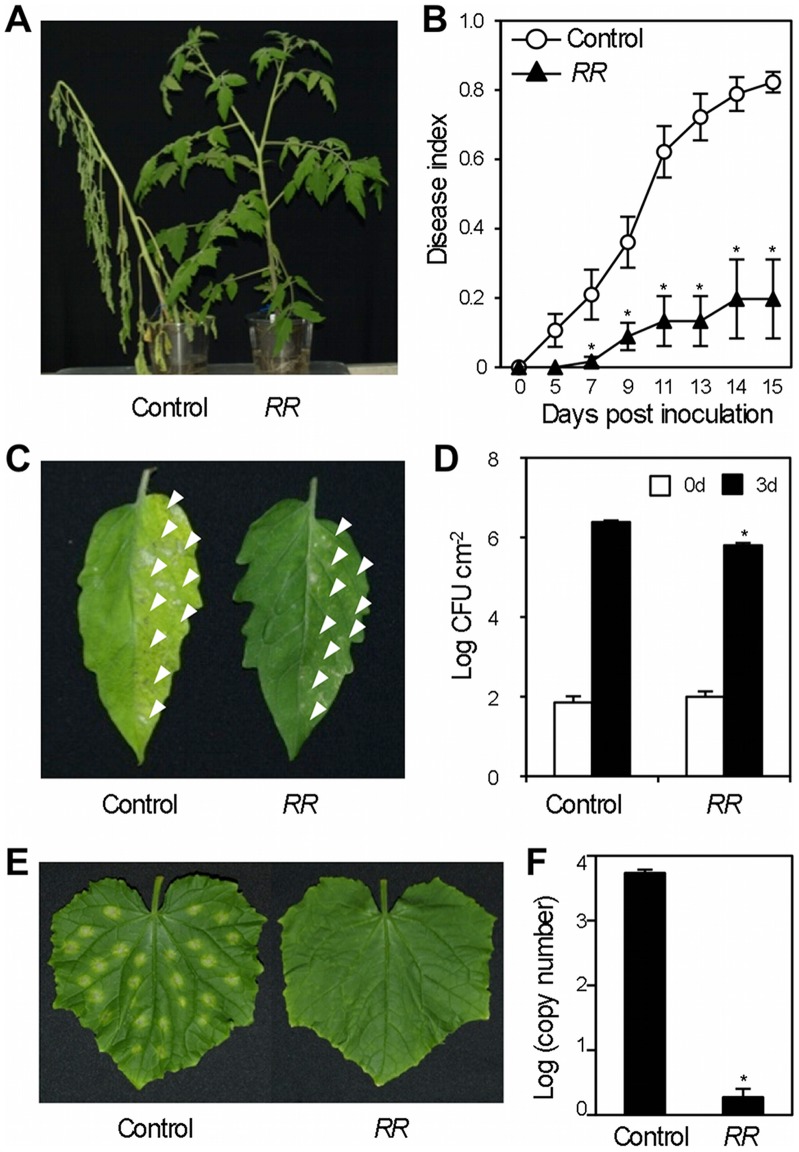
Transformation with *RPS4*/*RRS1* breaks restricted taxonomic functionality. (A,B) *R. solanacearum* resistance analysis in *RPS4*/*RRS1* dual *R* gene-transformed tomato (*RR*) and control plants. Two independent T2 transgenic lines carrying both *RPS4* and *RRS1* have been tested. Six-week-old tomato plants were inoculated with *R. solanacearum* expressing *popP2*. (A) Disease symptoms on tomato plants inoculated with *R. solanacearum* expressing *popP2* at 15 dpi. (B) Plants were rated every other day on a 0 to 5 disease index scale from 0 (no visible wilt) to 5 (the whole plant is dead). Each point represents the mean disease index (± SE) for three independent experiments, each containing 5 to 10 plants per treatment. The asterisks indicate statistical significance from the controls (Dunnett’s method, *P*<0.05). The control plants (vector control) wilted after inoculation with *R. solanacearum* expressing *popP2,* while *RPS4*/*RRS1* transformed plants (*RR*) were resistant. (C,D) Infection assays with *Pseudomonas syringae* pv. *tomato* DC3000 carrying the effector AvrRps4 (*Pst-avrRps4*) in *RPS4*/*RRS1* dual *R* gene-transformed tomato (*RR*) and control plants. Two independent T2 transgenic lines carrying both *RPS4* and *RRS1* were tested. The right sides of leaves of six-week-old tomato plants were infiltrated with bacterial suspensions (5×10^4^ cfu ml^−1^). There were 10–12 inoculation sites per leaf. Inoculation sites indicated by arrowheads. (C) Disease symptoms on tomato leaves inoculated with *Pst-avrRps4* at 7 dpi. (D) Leaves were harvested at 0 and 3 dpi. Leaves infected with *Pst*-*avrRps4* developed chlorotic lesions at 3 dpi. Bars indicate SE (*n* = 6). The asterisk indicates statistical significance from the control (3d) (Dunnett’s method, *P*<0.05). The experiment was repeated at least two times with similar results. (E,F) *Colletotrichum orbiculare* resistance in *RPS4*/*RRS1* dual *R* gene-transgenic cucumber (*RR*) and control plants. Four independent T2 transgenic lines carrying both *RPS4* and *RRS1* were tested. (E) Mature leaves of 2.5 true leaf stage seedlings were inoculated with spotting 5 µl of a conidial suspension of *C. orbiculare* (5×10^5^ spores ml^−1^) on the leaf. Photographs were taken at 6 dpi. (F) Mature leaves of 2.5 true leaf stage seedlings were spray-inoculated with a conidial suspension of *C. orbiculare* (5×10^5^ spores ml^−1^). Pathogen growth was determined by measuring *C. orbiculare-actin* mRNA by qRT-PCR. Bars indicate SE. The asterisk indicates statistical significance from the control (Dunnett’s method, *P*<0.05). The experiment was repeated at least two times with similar results.

To test whether the *RPS4*/*RRS1* pair confer immunity in another solanaceous plant, we generated transgenic tomato plants. Seven transgenic tomato T1 plants carrying a 10.9 kbp genomic fragment containing *RPS4* and *RRS1* were obtained and shown to contain these transgenes by PCR. T2 progenies derived from selfing T1 plants were assessed for the presence of the transgenes by PCR (Figure S1). Expression of the transgenes in T2 plants was confirmed by qRT-PCR (Figure S2). The bacterial wilt phytopathogen *R. solanacearum* is a serious soilborne disease that attacks almost 200 plant species in 33 plant families, including Solanaceae. Tomato plants (*Solanum lycopersicum*) transformed with *RPS4* and *RRS1* were resistant to *R. solanacearum* expressing *popP2* (Figure 4A, B), but susceptible to *R. solanacearum* without *popP2*, indicating that the conferred resistance is specific for the PopP2 effector (Figure S6A). *Pseudomonas syringae* pv. *tomato* DC3000 (*Pst*) causes bacterial speck on tomato, a disease characterized by defoliation, blossom blight, and lesions on developing fruit. Transgenic tomato plants were resistant to *Pst-avrRps4* (Figure 4C, D) but not to *Pst* containing a vector control (Figure S6B), indicating that conferred resistance is specific for the AvrRps4 effector. In addition, two independent T2 progenies segregated 3∶1 for resistance versus susceptibility to *R. solanacearum* expressing *popP2* and *Pst-avrRps4*. These transgenic plants grew normally and showed no significant constitutive expression of inducible defense-related gene *PR1* (Figure S3D & S4).

### Transgenic Cucumber Plants Expressing *RPS4* and *RRS1* are Resistant to *Colletotrichum orbiculare*

To investigate if the *RPS4*/*RRS1* pair confers broad-range resistance, we tested *Colletotrichum orbiculare* (*C. orbiculare*) on cucumber (*Cucumis sativus*; Cucurbitaceae) (*C. sativus*). Six transgenic cucumber T1 plants carrying a 10.9 kbp genomic fragment containing *RPS4* and *RRS1* were obtained. T2 progenies derived from selfing T1 plants were assessed for the presence of the transgenes by PCR (Figure S1). Expression of the transgenes in T2 plants was confirmed by qRT-PCR (Figure S2). Wild type cucumber plants developed brown necrotic lesions surrounded by a yellow halo, a typical symptom of anthracnose disease (Figure 4E). In contrast, *RPS4*/*RRS1* cucumber plants were highly resistant, developing only small necrotic flecks at the inoculated sites, indicative of an active defense reaction (Figure 4E, F). In addition, four independent T2 cucumber progenies segregated 3∶1 for resistance versus susceptibility to *C. orbiculare*. As in *N. benthamiana* and tomato, introduction of the *RPS4*/*RRS1* pair did not induce autoimmunity, indicating that *RPS4* and *RRS1* are tightly regulated in cucumber (Figure S3E & S4). *RPS4*/*RRS1* thus confers resistance to *Colletotrichum* sp. in taxonomically distinct families.

## Discussion

We demonstrate here that the *RPS4*/*RRS1 R* gene pair from *Arabidopsis* functions in other Brassicaceae plants, *B. rapa* and *B. napus*, as well as in *N. benthamiana*, tomato and cucumber. This provides that transfer of NB-LRR-type *R* genes confers resistance to multiple pathogens in taxonomically distinct families. The transfer of either *RPS4* or *RRS1* failed to provide resistance in transgenic plants (Figure 2A, B & 3), thus the success of interfamily transfer is likely due to the “dual *R*” system.

The number of known pairs of *R* genes is increasing [11]. For example, the *R* gene pairs, *RPP2A*/*RPP2* [12], *N*/*NRG1* [13], *RPM1/TAO1* [14], *Lr10*/*RGA2* [15], *Pi5-1*/*Pi5-2* [16], *Pikm1-TS*/*Pikm2-TS* [17], are only functional when both genes are present and we postulate that some of those pairs may also function in taxonomically distinct families when expressed together. It is also possible that some apparently singular *R* genes may require a complement to function. The mechanism of how these “dual R” proteins work together is unknown. However, one of the potential functions of the pair is as a negative regulator, as expression of single *R* genes often leads to inappropriate autoimmune responses in a taxonomically distinct family where no partner is available [18]. In this sense, expression of R proteins along with the corresponding proteins that are the target of pathogen effectors (often called ‘guardee’) may also be able to overcome restricted taxonomic functionality, since loss of the guardees often triggers R protein activation [1], [6].

Because RPS4 and RRS1 specifically react to bacterial effectors AvrRps4 and PopP2 in both Brassicaceae and Solanaceae plants, the recognition mechanism should be highly conserved across species, or built in the R protein pair itself. Recent reports indicate that AvrRps4 directly targets Enhanced Disease Susceptibility 1 (EDS1), but does not interact with RPS4 [19], [20]. EDS1 is a highly conserved key factor in TIR-NB-LRR-mediated ETI and interacts with several TIR-NB-LRR protiens, including RPS4 [21]. However, more recent report by Sohn et al. (2012) showed that AvrRps4 and EDS1 do not directly interacted [22]. Thus, EDS1 can be the direct or indirect target of AvrRps4 in both Brassicaceae and Solanaceae plants. If this is the case, RPS4 and RRS1 could confer broad resistance, as many pathogens would target EDS1 as the key immunity protein. Unlike the AvrRps4-RPS4/RRS1 example, PopP2, a YopJ-like family of cysteine proteases, directly binds to RRS1 [23]. As PopP2-dependent immune response requires RPS4 (Figure 3), it is possible that RPS4 can recognize the interaction between PopP2 and RRS1. In any case, PopP2 produced by *Ralstonia* could be detected in many plants by introgressing the RPS4/RRS1 cassette.

Perhaps the biggest surprise to us is that the RPS4/RRS1 pair confers immunity against both *C. higginsianum* and *C. orbiculare* in taxonomically distinct plant families. This implies that the target (*i.e.* host immunity-related proteins) of the unidentified *Colletotrichum* effectors is conserved in Brassicaceae and Cucurbitaceae, and/or that the effectors recognized by RPS4/RRS1 are highly conserved in *Colletotrichum*. As is the case for AvrRps4 and PopP2, *Colletotrichum* effectors may directly target EDS1 or RRS1. The sequenced *Colletotrichum* genomes do not contain apparent AvrRps4 and PopP2 homologs, suggesting that the targeting mechanism should be distinct from the bacterial targeting mechanism [24]. There are a number of potential highly conserved effectors in *Colletotrichum* spp., and some of them could be detected by the RPS4/RRS1 pair by an unknown mechanism. Isolation of the *Colletotrichum* effectors would resolve the problem. In any case, RPS4/RRS1 could provide resistance to *Colletotrichum* species among a number of agronomically important crops and vegetables.

Our study demonstrates that NB-LRR-type *R* gene-based immunity can be transferred to distantly related species once the right gene pair is identified. Each plant genome contains about 150 to 500 NB-LRR-type *R* genes [25], [26], some of which are likely to form pairs. As in the case of *RPS4/RRS1*, some gene pairs might provide a wide range resistance to multiple pathogens. Thus our finding provides a new strategy for creating pathogen-resistant vegetables and crops by a transgenic approach using previously unexploited resource of genetic resistance.

## Materials and Methods

### Plant Materials

*Brassica rapa* L. Perviridis Group (Japanese Mustard Spinach, Komatsuna cv. Osome, Takii & Co., Ltd., Kyoto, Japan), *B. napus* L. cv. Westar plants, tobacco (*N. benthamiana*), tomato (*Solanum lycopersicum* L. cultivar Moneymaker) and cucumber (*C. sativus* L. cultivar Shinhokusei No. 1) plants were grown in Soil-mix (Sakata Seed Corp., Yokohama, Japan) and expanded vermiculite (1.5–2 mm granules) at a ratio of 1∶1 in a growth chamber at 22°C with 12-h light for *B*. *rapa*, *B*. *napus* and cucumber, at 25°C with 16-h light for *N. benthamiana*, and at 25°C during the daylight hours for tomato.

### Construction of the *R* Gene Plasmid

A 10.9 kbp genomic fragment containing *RPS4* and *RRS1* was amplified from *A. thaliana* ecotype Wassilewskija (Ws-0) genomic DNA by PCR using RR1 primers listed in Table S1, and cloned into pCR8GW-TOPO (Life Technologies, CA, USA). The resultant plasmid, pCR8GW-RR-Ws, was subcloned into the destination vector pBI-GW-NOS (Inplanta Innovations Inc., Yokohama, Japan) using the LR cloning reaction. The 6.3 kbp genomic *RPS4* fragment, including approximately 2.1 kbp upstream and 109 bp downstream regions, was cloned into pBI101-SK+ [7]. The 8.2 kbp genomic *RRS1* fragment, including approximately 1.8 kbp upstream and 176 bp downstream regions, was subcloned into the destination vector pGWB1 using the LR cloning reaction [7].

### Transformation

*B. rapa* and *B. napus* were transformed by inoculating hypocotyl sections with *Agrobacterium tumefaciens* (*A. tumefaciens*) (*Rhizobium radiobactor*) strain EHA101 harboring the binary vector containing the fragments described above [27], [28]. *N. benthamiana* transformation was by the leaf disk method using *A. tumefaciens* strain LBA4404 harboring binary vector containing the fragments described above [29], [30]. Transformation of tomato was performed according to the cotyledon explant method using *A. tumefaciens* strain C58C1Rif^R^ (pGV2260) harboring the binary vector and genomic *RPS4/RRS1* fragment described above [31]. Transformation of cucumber was by the cotyledon explant method using *A. tumefaciens* strain EHA105 harboring the binary vector with the genomic *RPS4/RRS1* fragment described above [32], [33].

### *C. higginsianum* Strain and Inoculations

*C. higginsianum* Saccardo isolates (MAFF305635) were obtained from the MAFF Genebank project, Japan. Mature leaves of 2.5 true leaf stage seedlings (12 h light cycle) were inoculated as described previously [7].

### Quantification of C. higginsianum Actin mRNA

Plants inoculated with *C. higginsianum* were harvested at 4 d post inoculation (dpi) for qRT-PCR analysis. The primers for qRT-PCR are listed in Table S1. Quantification of *C. higginsianum* was performed as described previously [34]. QRT-PCR data for *C. higginsianum* actin (*Chin-ACT*) and *Br-CBP20* expression from *B*. *rapa*, or *Chin-ACT* and *B*. *napus actin* (*Bn-ACT*) expression from *B*. *napus* were obtained from a standard curve of cycle times as a function of copy number. The abundance of *Chin-ACT* was normalized with *Br-CBP20* or *Bn-ACT* in infected samples.

### Transient Expression Assay in *N. benthamiana*

The *avrRps4* and *popP2* DNA fragments were amplified from *Pst-avrRps4* and *Ralstonia solanacearum* strain RS1002 genomic DNA, respectively, by PCR using avrRps4 and popP2 primers listed in Table S1, and cloned into pCR8GW-TOPO. The pSfinx vector [35] was converted into a Gateway destination vector using the Gateway Vector Conversion System (Life Technologies). A Gateway Reading Frame Cassette B was introduced into the original cloning cassette of the pSfinx vector using the *Sma*I site. The *avrRps4* and *popP2* DNA fragments were cloned into the modified pSfinx vector using the LR cloning reaction (pSfinx*-avrRps4* and pSfinx-*popP2*, respectively). *N. benthamiana* plants were grown in a growth chamber at 25°C with 16-h light. Plants at the age of four-week-old were used in the experiment. Overnight bacterial cultures of *A. tumefaciens* strain GV3101 (pMP90, pSoup) containing pSfinx*-avrRps4* or pSfinx-*popP2* were harvested by centrifugation. Cells were washed three times in induction buffer (10 mM MES, pH 5.6, 10 mM MgCl_2_ and 150 µM acetosyringone), re-suspended in induction buffer to an OD_600_ of 0.5, and incubated for 2 h at room temperature. *Agrobacterium* cells were hand-infiltrated into *N. benthamiana* leaves with a 1 ml syringe. Cell death responses began to appear on infiltrated leaves 5 d after infiltration.

### Infection of Tomato with *R. solanacearum*

*R. solanacearum* strains RS1002 and RS1002*-ΔpopP2* were described previously [7], [36]. *R. solanacearum* was streaked on tetrazolium medium (TTC) [37] and incubated at 28°C for 2 d. A single colony was grown in CPG liquid medium [38] at 28°C for 24 h. Bacterial cells were harvested and suspended with distilled water to an OD_600_ of 0.1 (about 1×10^8^ cfu ml^−1^). Tomato cultivar Moneymaker (susceptible to *R. solanacearum* RS1002) plants were grown on 9 cm-diameter Jiffy peat pots containing Soil-mix and expanded vermiculite at the ratio of 1∶1 in a greenhouse at 24 to 25°C. Six-week-old seedlings at approximately the eight- to nine-leaf stage were inoculated by drenching the plant saucer near the root of each plant with 30 ml of the bacterial suspension. Plant roots were not wounded before inoculation. Observations for bacterial wilt symptoms were carried out at 5, 7, 9, 11, 13, 14 and 15 dpi according to the following scale: 0, no visible wilt; 1, one or two leaves wilted; 2, three or four leaves wilted; 3, five or six leaves wilted; 4, seven to nine leaves wilted; and 5, more than ten leaves wilted. The disease index (DI) was calculated as DI = Σ(grade × number of wilting plants)/(5× number of total plants) [39].

### Infection of Tomato with *Pst* and *Pst*-*avrRps4*

*Pst* and *Pst*-*avrRps4* [8], [40] were grown in liquid King’s B medium containing kanamycin (25 µg ml^−1^) and rifampicin (25 µg ml^−1^). Bacteria were harvested by centrifugation, cell pellets were washed with 10 mM MgSO_4_, and resuspended in 10 mM MgSO_4_ to a concentration of 5×10^4^ cfu ml^−1^ for *in planta* growth assays. Tomato plants (susceptible to both *Pst* and *Pst*-*avrRps4*) were grown as above. Six-week-old seedlings at approximately the eight- to nine-leaf stage were used for virulence assays. Plants were transferred to a growth chamber with 95–100% humidity at 25°C with 12 h light 1 d prior to inoculation by infiltration. Abaxial leaf surfaces of tomato plants were infiltrated with the bacterial suspension with a 1 ml syringe. Bacterial growth was determined 0 and 3 d after infiltration using five leaf disks, as previously described [40].

### *C. orbiculare* Inoculations

*C. orbiculare* strain 104-T [41] was maintained on potato dextrose agar (Difco, MI, USA) at 24°C in the dark. Conidia were obtained by gently scraping 7 d cultures as described [42]. Mature leaves of 2.5 true leaf stage seedlings were used for virulence assays. Quantification of *C. orbiculare* was performed by spotting 5 µl of a conidial suspension (5×10^5^ conidia ml^−1^ in distilled water) on the surface of detached cucumber leaves, and then five leaf-disks were cut using a cork borer (No. 3) at 6 dpi. Leave disks were frozen in liquid nitrogen and ground using an SH-48 grinding apparatus (Kurabo, Osaka, Japan). Preparation of total RNA and first-strand cDNA, qRT-PCR were performed according to a method described previously [34]. Nucleotide sequences of *C. orbiculare actin* (*Cor-ACT*) primers are listed in Table S1. QRT-PCR data for *Cor-ACT* and *C. sativus EF1α* (*Cs-EF1α*) expression from cucumber were collected as copy number obtained from a standard curve of cycle times. The abundance of *Cor-ACT* was normalized against *Cs-EF1α* in infected samples.

### Expression Analysis of Defense-related Gene in Transgenic Plants Expressing *RPS4* and *RRS1*

Expression of the *pathogenesis-related 1* (*PR1*) gene involved in the plant defense responses was determined by qRT-PCR of RNA from five leaf-disks cut from leaves of the 2.5 true leaf stage T2 transgenic *B. rapa*, *B. napus* and cucumber, four-week-old T3 transgenic *N. benthamiana*, and three-week-old T2 transgenic tomato plants carrying both *RPS4* and *RRS1* using a cork borer (No. 3). Total RNA was isolated and treated with RNase-free DNase (Promega, WI, USA). 500 ng of total RNA was synthesized with oligo dT primer using a PrimeScript RT reagent kit (Takara, Otsu, Japan). QRT-PCR was performed with SYBR Green PCR Master Mix (BIO-Rad Laboratories, CA, USA) using the first-strand cDNA as a template on an MJ Opticon (Bio-Rad Laboratories). QRT-PCR mixtures consisted of 1xSYBR Green I PCR Master Mix and 200 nM (each) sense and antisense primers. Following a preliminary denaturation step at 95°C for 30 s, the reaction mixtures were cycled 40X at 95°C for 5 s and at 65°C for 20 s. The target sample copy number was averaged for two reactions, and the experiment was repeated twice. Expression of the *Br-CBP20* for *B*. *rapa*, *Bn-ACT* for *B*. *napus*, *EF1α* for *N. benthamiana* and cucumber, or *Tip41* for tomato was used for normalization. *PR1* gene expression is shown as relative values set at a value of 1 in the control plants. Nucleotide sequences of gene-specific primers for each gene are listed in Table S1. Bars indicate SE. This experiment was repeated twice with similar results.

## Supporting Information

Figure S1 **Diagram of** ***RPS4*** **and** ***RRS1*** **genes inserted into a binary vector.** (A) The 6.3 kbp genomic *RPS4* fragment, including approximately 2.1 kbp upstream and 109 bp downstream regions, (B) the 8.2 kbp genomic *RRS1* fragment, including approximately 1.8 kbp upstream and 176 bp downstream regions, and (C) the 10.9 kbp genomic fragment containing both *RPS4* and *RRS1* were cloned into binary vector pBI101-SK+ [7], pGWB1 [7], and pBI-GW-NOS, respectively. Arrowboxes indicate *RPS4* and/or *RRS1* genome fragments. Lines indicate the polylinker regions of binary vector. Transgenic plants were assessed for the presence of the transgene by PCR. Locations of primers used for PCR are indicated by arrows. Specific PCR primers: KY and N3 for the polylinker of binary vector, RPS4-SF5 and NRS2-S4 for *RPS4* gene, RRS1-S10-2 and RPS4-SF2 for *RRS1* gene. The primers for qRT-PCR are listed in Table S1.
(TIF)

Figure S2 **Expression of the transgene** ***RPS4*** **and** ***RRS1*** **in transgenic plants.** Five leaf disks were cut from leaves of the 2.5 true leaf stage control and transgenic *B. rapa* (T2), *B. napus* (T2), *N. benthamiana* (T3), tomato (T2) and cucumber (T2) plants carrying both *RPS4* and *RRS1* (*RR*), either *RRS1* or *RPS4* alone, and total RNA was isolated for qRT-PCR analysis. Expression of the transgene *RPS4* and *RRS1* in transgenic plants was quantified by qRT-PCR. Bars indicate SE. The experiment was repeated twice with similar results. The primers used here are specific to the transgenes (*Arabidopsis RPS4* and *RRS1*). As expression of *RPS4* was slightly detected in *B. napus* wild-type plants, we assume *B. napus* has the homologue of *Arabidopsis RPS4* or non-specific binding of the primers occurred for transcripts of *B. napus.*
(TIF)

Figure S3 **Growth of transgenic plants expressing** ***RPS4*** **and** ***RRS1*** **.** Each picture shows four-week-old T2 transgenic *B. rapa* (A), three-week-old T2 transgenic *B. napus* (B), six-week-old T3 transgenic *N. benthamiana* (C), four-week-old T2 transgenic tomato (D), four-week-old T2 transgenic cucumber (E) carrying both *RPS4* and *RRS1* (*RR*) and control plants. The experiment was repeated more than three times with similar results.
(TIF)

Figure S4 **Expression of defense-related gene** ***PR1*** **in transgenic plants under normal growth conditions.** Five leaf disks were cut from leaves of the 2.5 true leaf stage T2 transgenic *B. rapa*, *B. napus* and cucumber, four-week-old T3 transgenic *N. benthamiana*, three-week-old T2 transgenic tomato carrying both *RPS4* and *RRS1* (*RR*) and control plants using a cork borer (No. 3). Total RNA was isolated for qRT-PCR analysis. *PR1* gene expression is shown as relative values set at 1 in the control plants. Bars indicate SE. The experiment was repeated twice with similar results.
(TIF)

Figure S5 **Quantification of** ***avrRps4*** **and** ***popP2*** **mRNA transiently expressed in transgenic** ***N. benthamiana*** **plants.** The fully expanded leaves of four-week-old T3 homozygous transgenic *N. benthamiana* expressing *RPS4* and/or *RRS1*, and control plants were infiltrated with *A. tumefaciens* strain GV3101 (pMP90, pSoup) containing pSfinx*-avrRps4* or pSfinx-*popP2.* Total RNA was isolated 24 h post inoculation. Expression of *avrRps4* and *popP2* in transgenic plants was quantified by qRT-PCR. Non: non infiltrated leaves. Bars indicate SE. The experiment was repeated twice with similar results.
(TIF)

Figure S6 **Growth of bacterial pathogens in** ***RPS4*** **and** ***RRS1*** **dual** ***R*** **gene-transformed tomato and control plants.** (A) *R. solanacearum* resistance analysis in *RPS4* and *RRS1* dual *R* gene-transformed tomato (*RR*). Six-week-old tomato plants were inoculated with *R. solanacearum* strain RS1002*-ΔpopP2*. Plants were rated every other day on a 0 to 5 disease index scale from 0 (no visible wilt) to 5 (the whole plant is dead). Each point represents the mean disease index (± SE) for three independent experiments, each containing 5 to 10 plants per treatment. Both control plants and transformants wilted after inoculation with *R. solanacearum* strain RS1002*-ΔpopP2.* (B) Infection assays with *Pseudomonas syringae* pv. *tomato* DC3000 (*Pst*) in *RPS4* and *RRS1* dual *R* gene-transformed tomato (*RR*). Leaves of six-week-old tomato plants were infiltrated with bacterial suspensions (5×10^4^ cfu ml^−1^). Leaves were harvested at 3 dpi. Growth of *Pst* in vector controls and dual *R* gene-transformed tomato had increased greatly by 3 dpi. Bars indicate SE. The experiment was repeated three times with similar results.
(TIF)

Table S1 **PCR primers used in this study.**
(TIF)
