# Supplementary figures and images for: Interfamily Transfer of Dual NB-LRR Genes Confers Resistance to Multiple Pathogens
Source: PLoS One. 2013 Feb 20;8(2):e55954. doi: 10.1371/journal.pone.0055954 (PMC3577827; doi:10.1371/journal.pone.0055954)

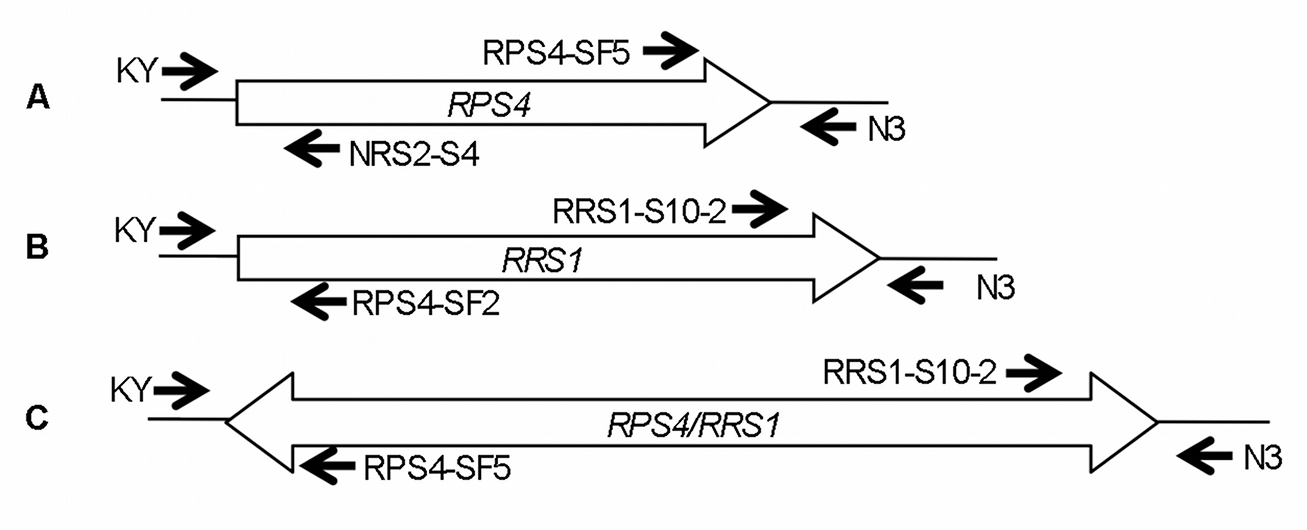

Supplement: Figure S1 — Diagram of RPS4 and RRS1 genes inserted into a binary vector. (A) The 6.3 kbp genomic RPS4 fragment, including approximately 2.1 kbp upstream and 109 bp downstream regions, (B) the 8.2 kbp genomic RRS1 fragment, including approximately 1.8 kbp upstream and 176 bp downstream regions, and (C) the 10.9 kbp genomic fragment containing both RPS4 and RRS1 were cloned into binary vector pBI101-SK+ [7], pGWB1 [7], and pBI-GW-NOS, respectively. Arrowboxes indicate RPS4 and/or RRS1 genome fragments. Lines indicate the polylinker regions of binary vector. Transgenic plants were assessed for the presence of the transgene by PCR. Locations of primers used for PCR are indicated by arrows. Specific PCR primers: KY and N3 for the polylinker of binary vector, RPS4-SF5 and NRS2-S4 for RPS4 gene, RRS1-S10-2 and RPS4-SF2 for RRS1 gene. The primers for qRT-PCR are listed in Table S1. (TIF) [file pone.0055954.s001.tif]

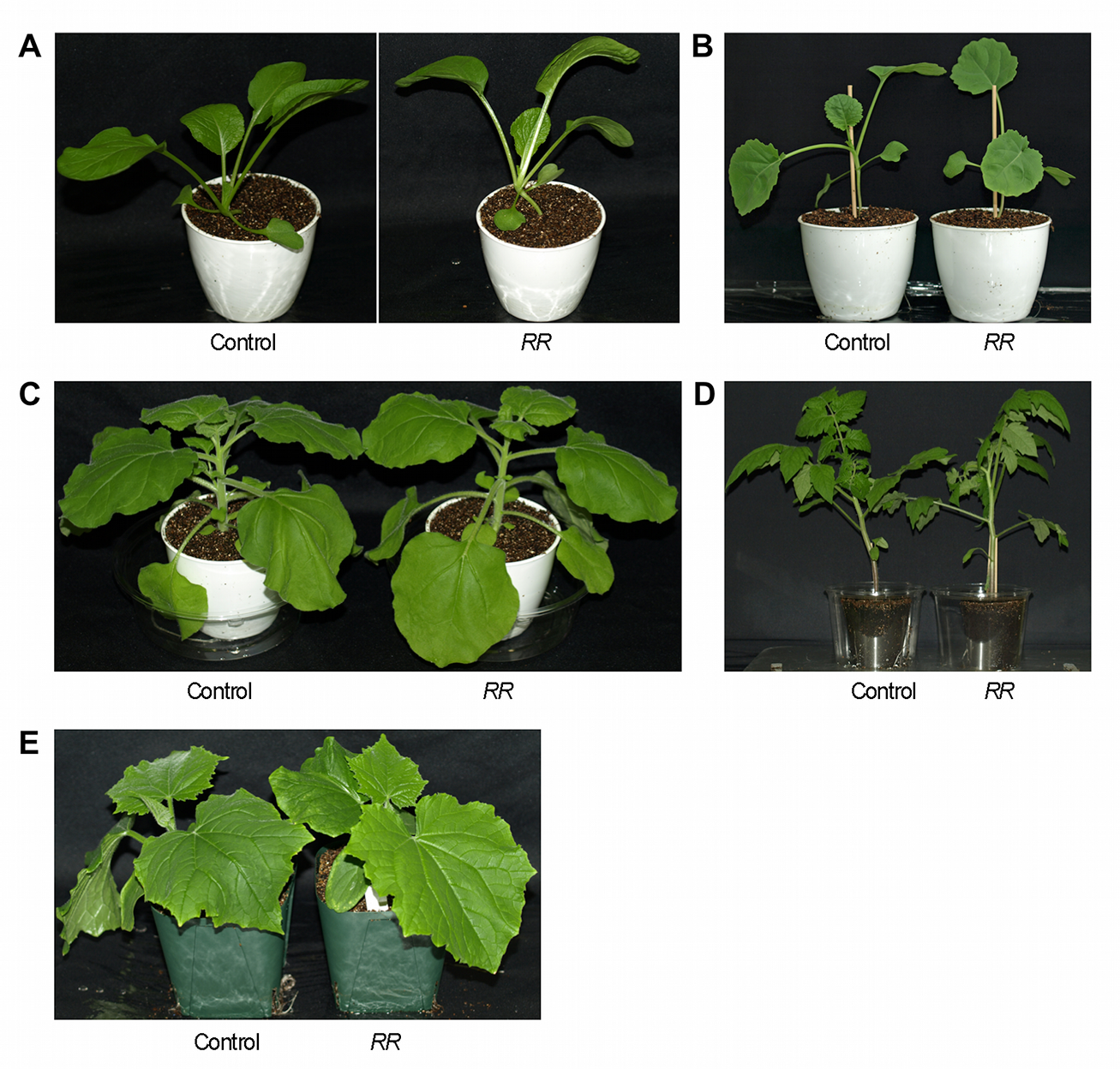

Supplement: Figure S3 — Growth of transgenic plants expressing RPS4 and RRS1 . Each picture shows four-week-old T2 transgenic B. rapa (A), three-week-old T2 transgenic B. napus (B), six-week-old T3 transgenic N. benthamiana (C), four-week-old T2 transgenic tomato (D), four-week-old T2 transgenic cucumber (E) carrying both RPS4 and RRS1 (RR) and control plants. The experiment was repeated more than three times with similar results. (TIF) [file pone.0055954.s003.tif]

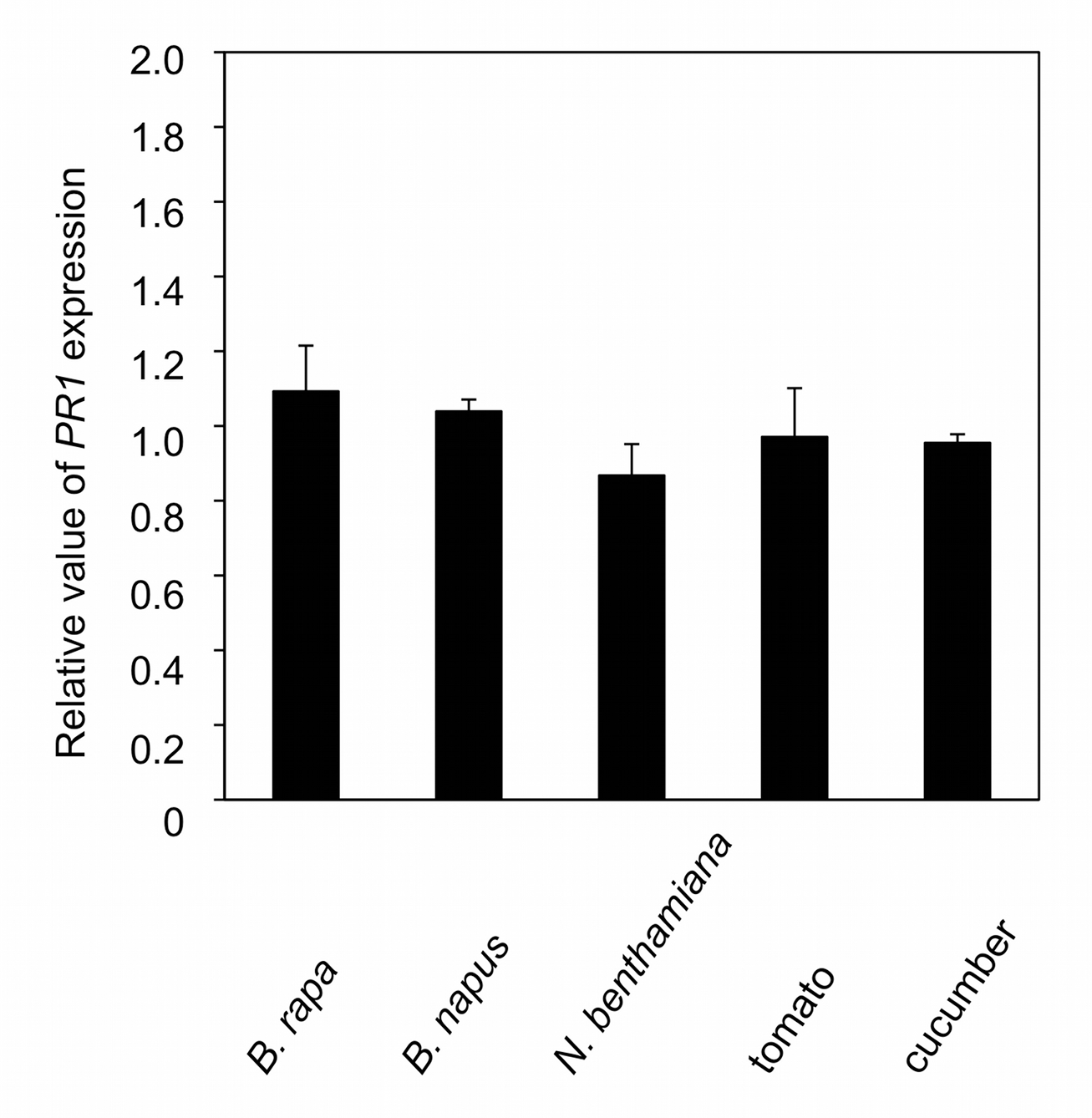

Supplement: Figure S4 — Expression of defense-related gene PR1 in transgenic plants under normal growth conditions. Five leaf disks were cut from leaves of the 2.5 true leaf stage T2 transgenic B. rapa, B. napus and cucumber, four-week-old T3 transgenic N. benthamiana, three-week-old T2 transgenic tomato carrying both RPS4 and RRS1 (RR) and control plants using a cork borer (No. 3). Total RNA was isolated for qRT-PCR analysis. PR1 gene expression is shown as relative values set at 1 in the control plants. Bars indicate SE. The experiment was repeated twice with similar results. (TIF) [file pone.0055954.s004.tif]

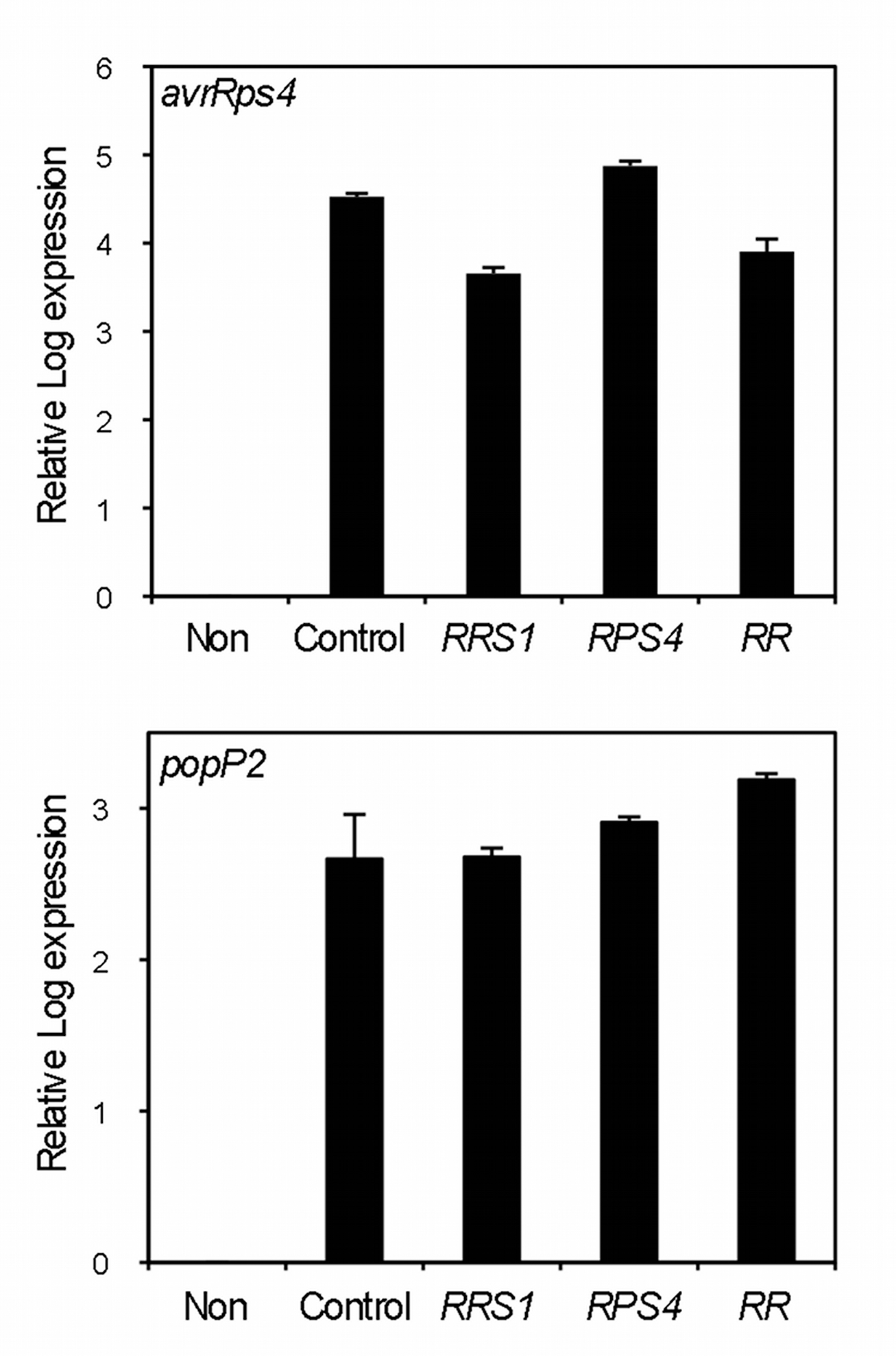

Supplement: Figure S5 — Quantification of avrRps4 and popP2 mRNA transiently expressed in transgenic N. benthamiana plants. The fully expanded leaves of four-week-old T3 homozygous transgenic N. benthamiana expressing RPS4 and/or RRS1, and control plants were infiltrated with A. tumefaciens strain GV3101 (pMP90, pSoup) containing pSfinx-avrRps4 or pSfinx-popP2. Total RNA was isolated 24 h post inoculation. Expression of avrRps4 and popP2 in transgenic plants was quantified by qRT-PCR. Non: non infiltrated leaves. Bars indicate SE. The experiment was repeated twice with similar results. (TIF) [file pone.0055954.s005.tif]

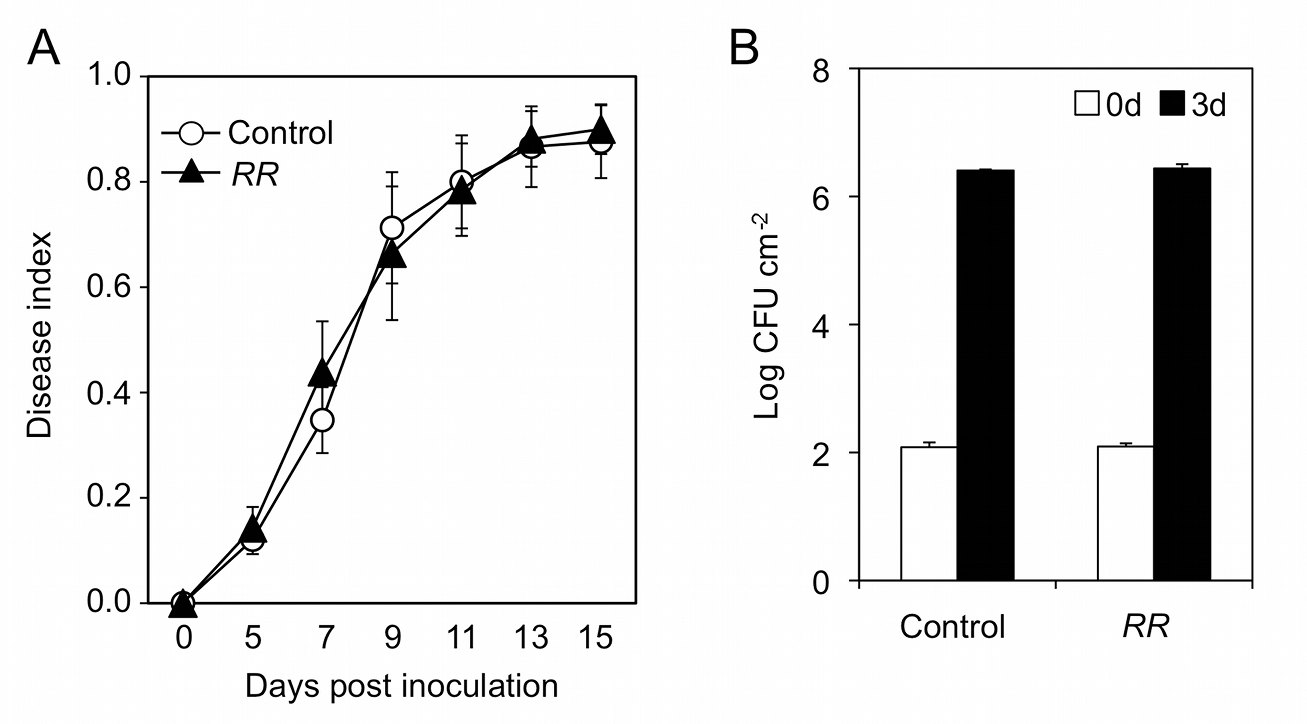

Supplement: Figure S6 — Growth of bacterial pathogens in RPS4 and RRS1 dual R gene-transformed tomato and control plants. (A) R. solanacearum resistance analysis in RPS4 and RRS1 dual R gene-transformed tomato (RR). Six-week-old tomato plants were inoculated with R. solanacearum strain RS1002-ΔpopP2. Plants were rated every other day on a 0 to 5 disease index scale from 0 (no visible wilt) to 5 (the whole plant is dead). Each point represents the mean disease index (± SE) for three independent experiments, each containing 5 to 10 plants per treatment. Both control plants and transformants wilted after inoculation with R. solanacearum strain RS1002-ΔpopP2. (B) Infection assays with Pseudomonas syringae pv. tomato DC3000 (Pst) in RPS4 and RRS1 dual R gene-transformed tomato (RR). Leaves of six-week-old tomato plants were infiltrated with bacterial suspensions (5×10^4^ cfu ml−1). Leaves were harvested at 3 dpi. Growth of Pst in vector controls and dual R gene-transformed tomato had increased greatly by 3 dpi. Bars indicate SE. The experiment was repeated three times with similar results. (TIF) [file pone.0055954.s006.tif]

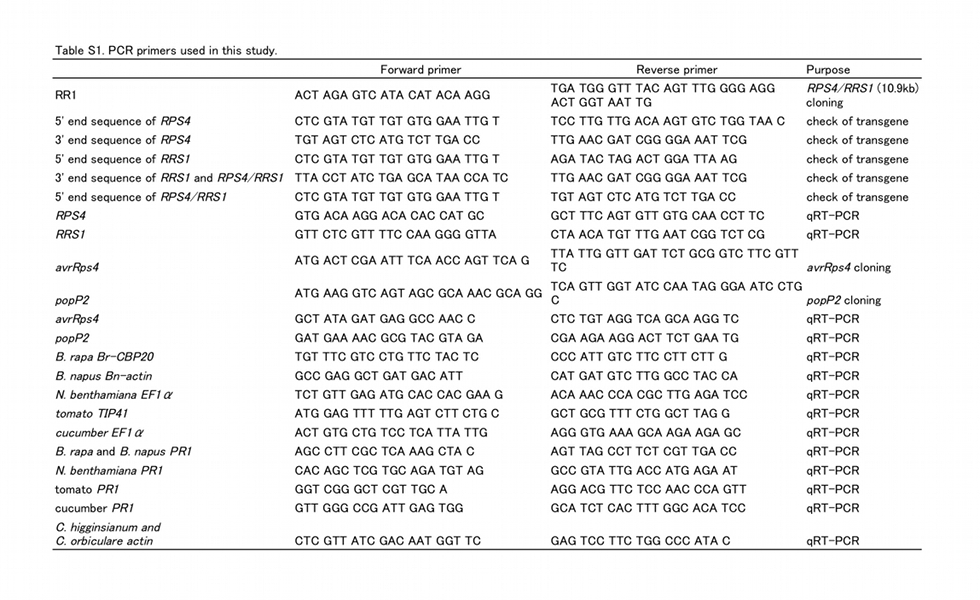

Supplement: Table S1 — PCR primers used in this study. (TIF) [file pone.0055954.s007.tif]
